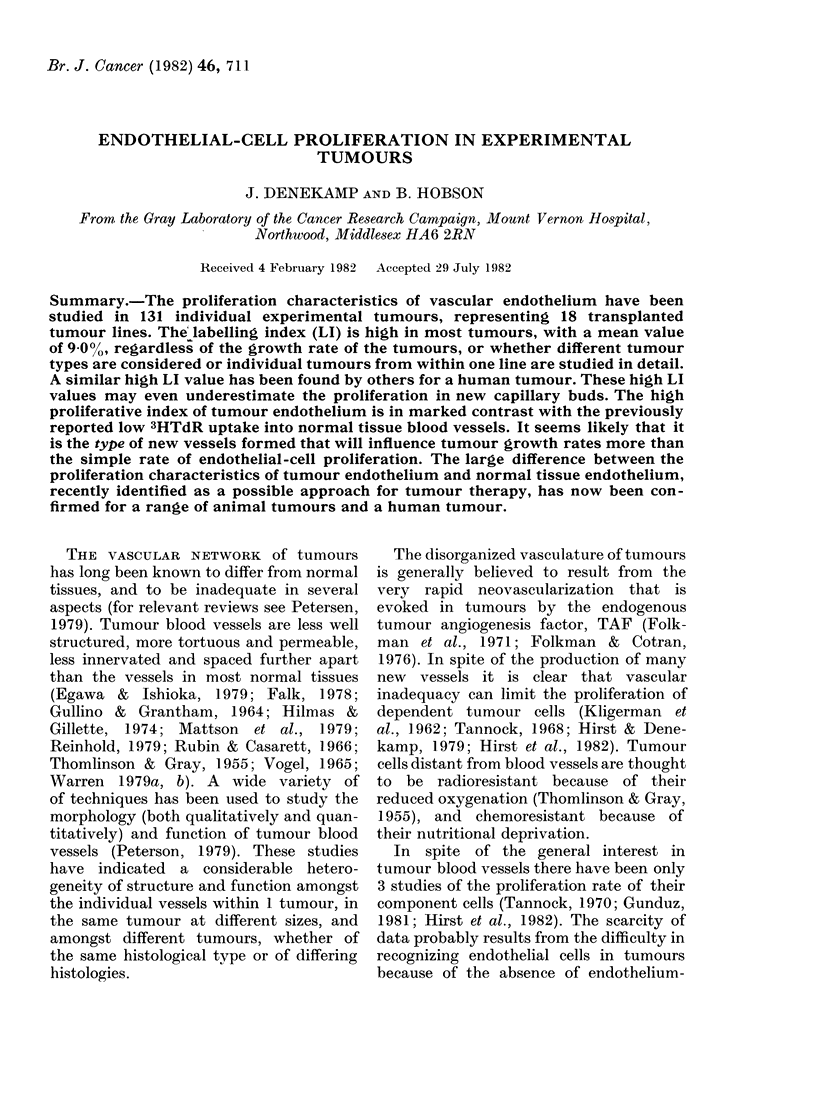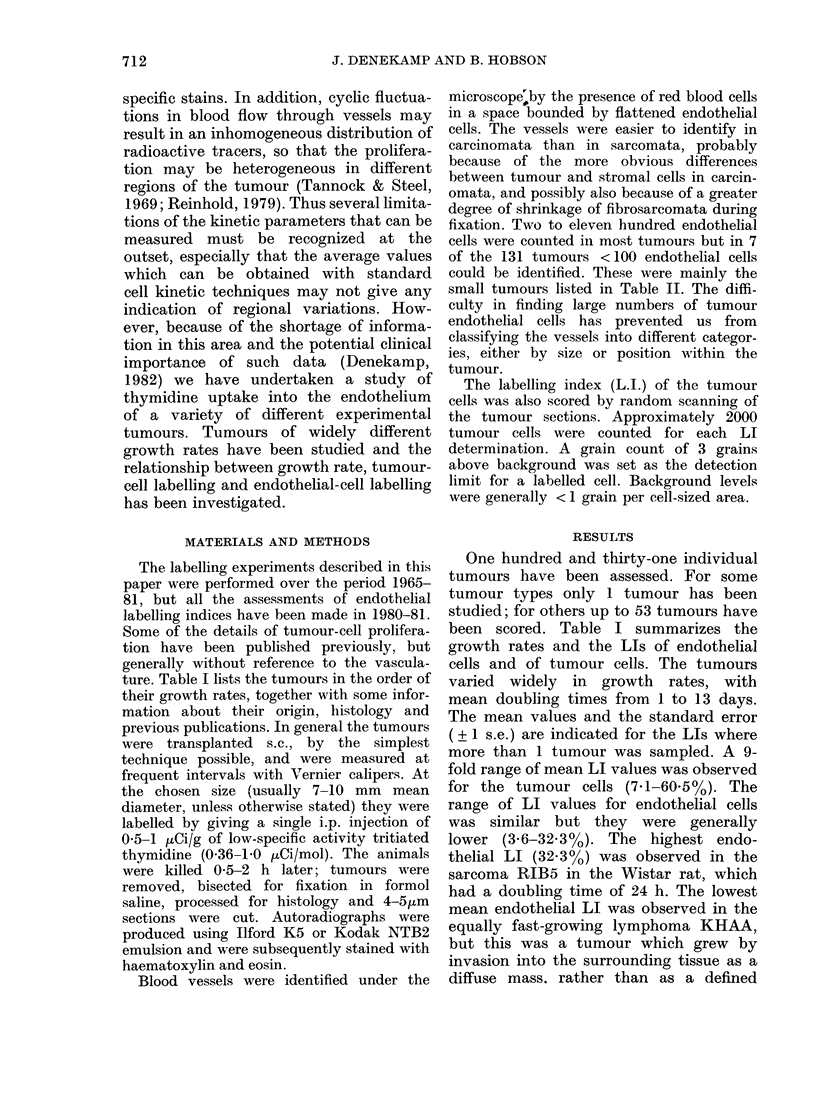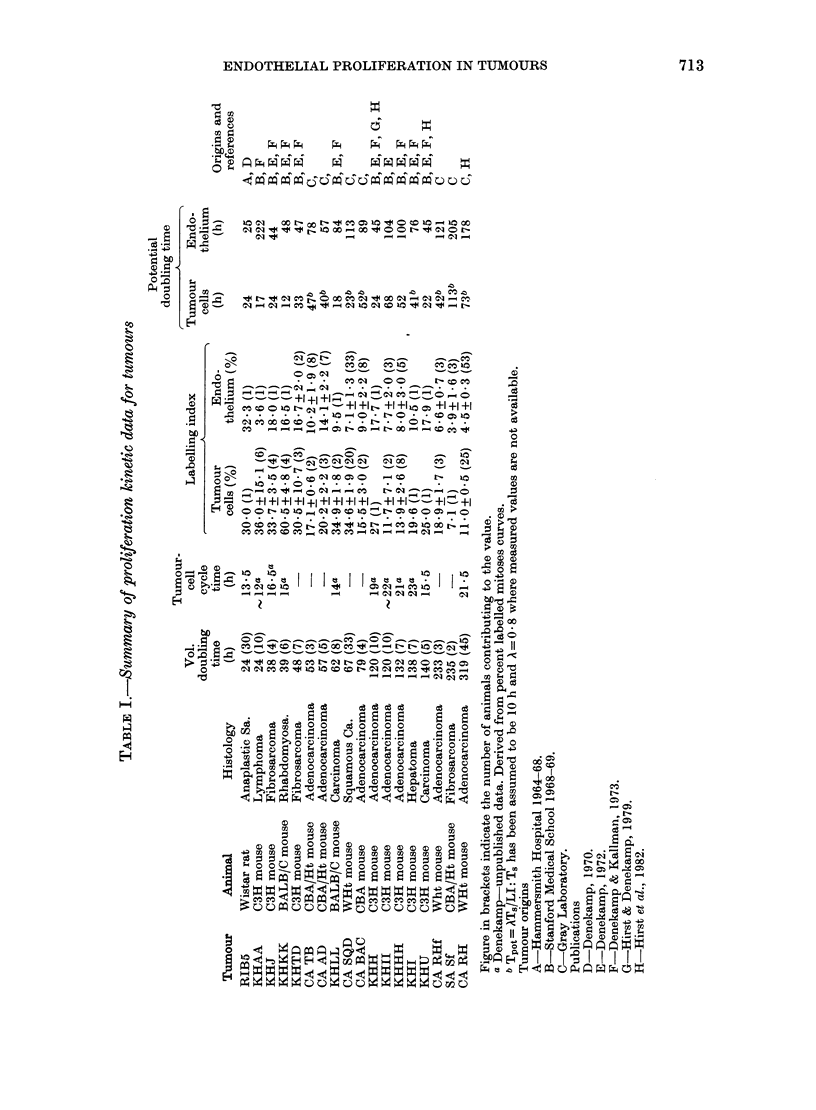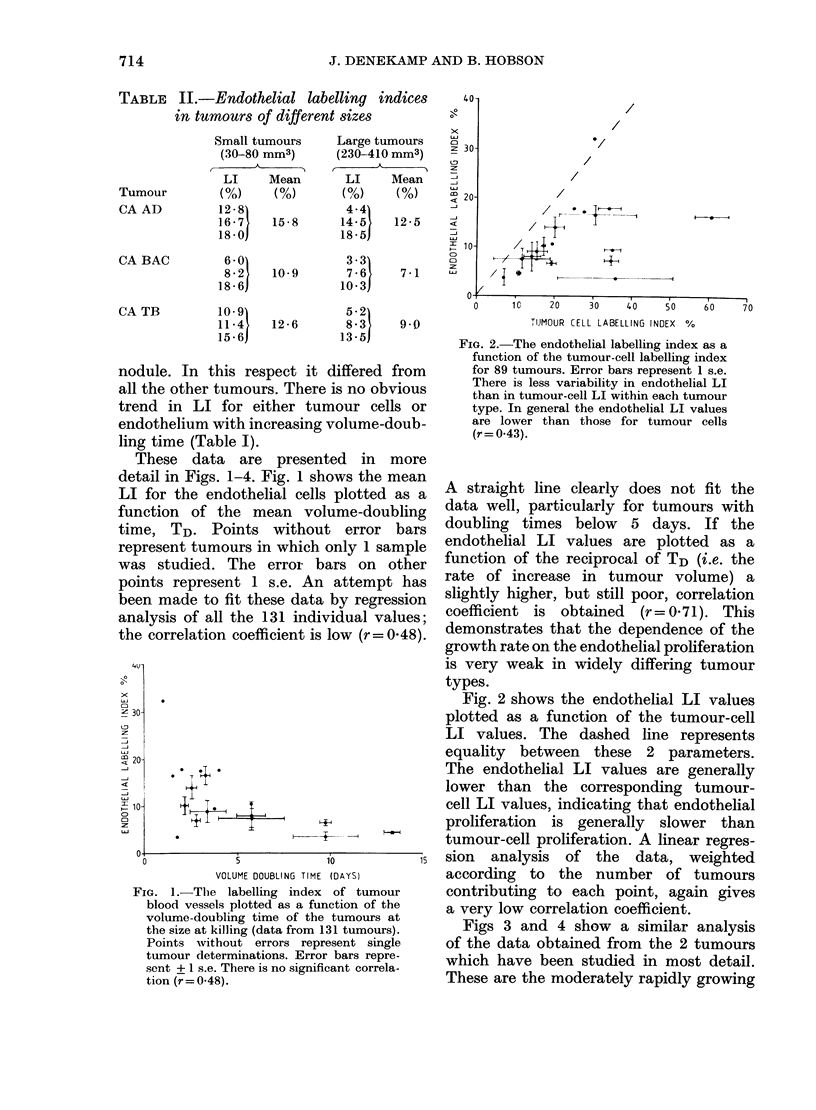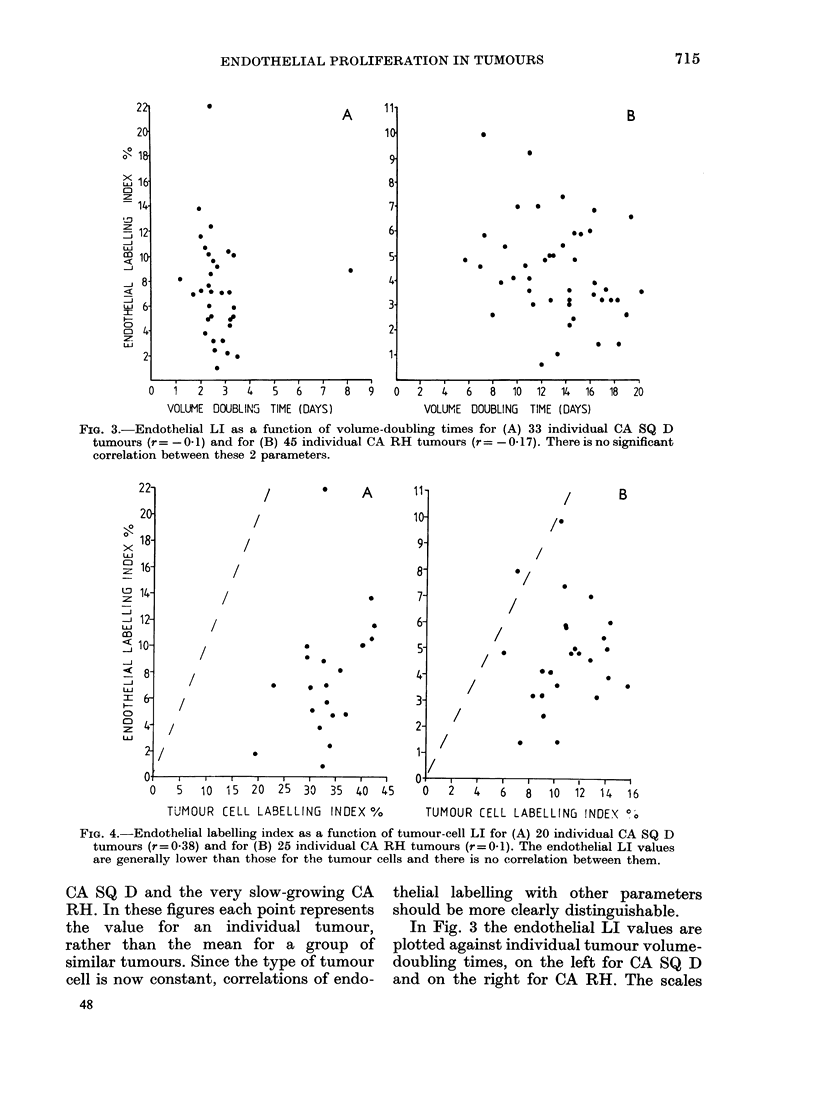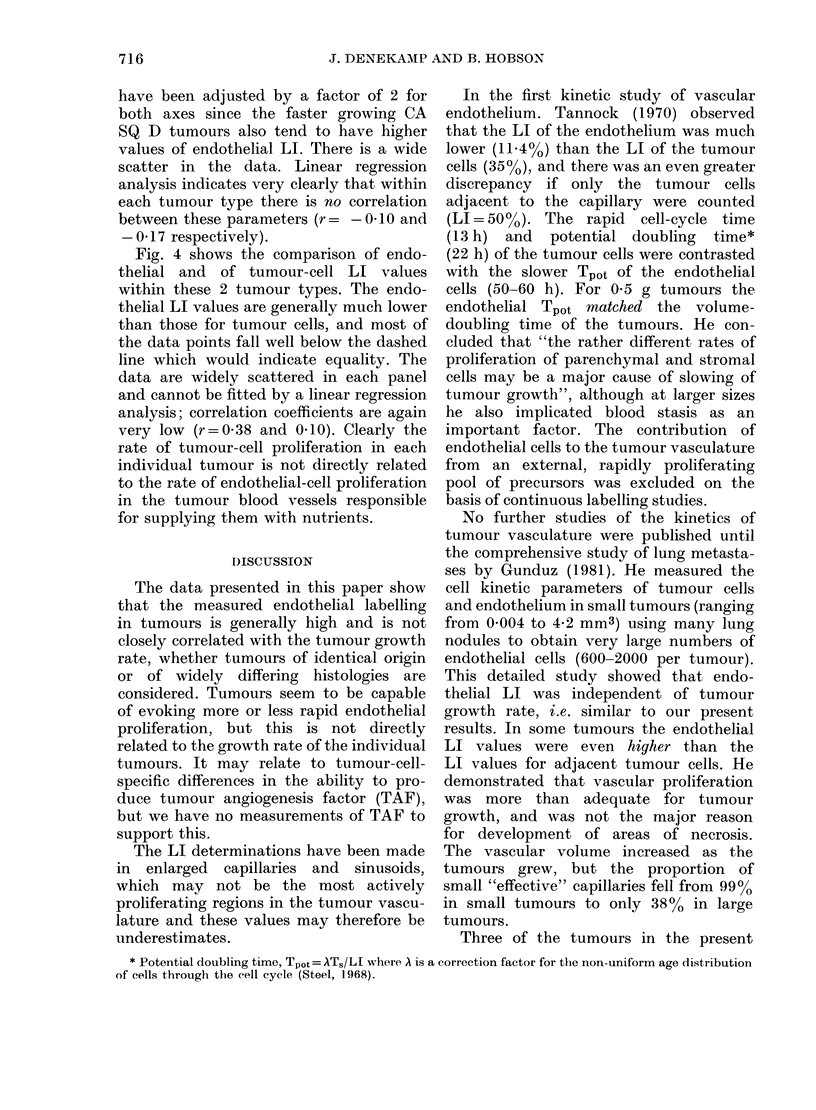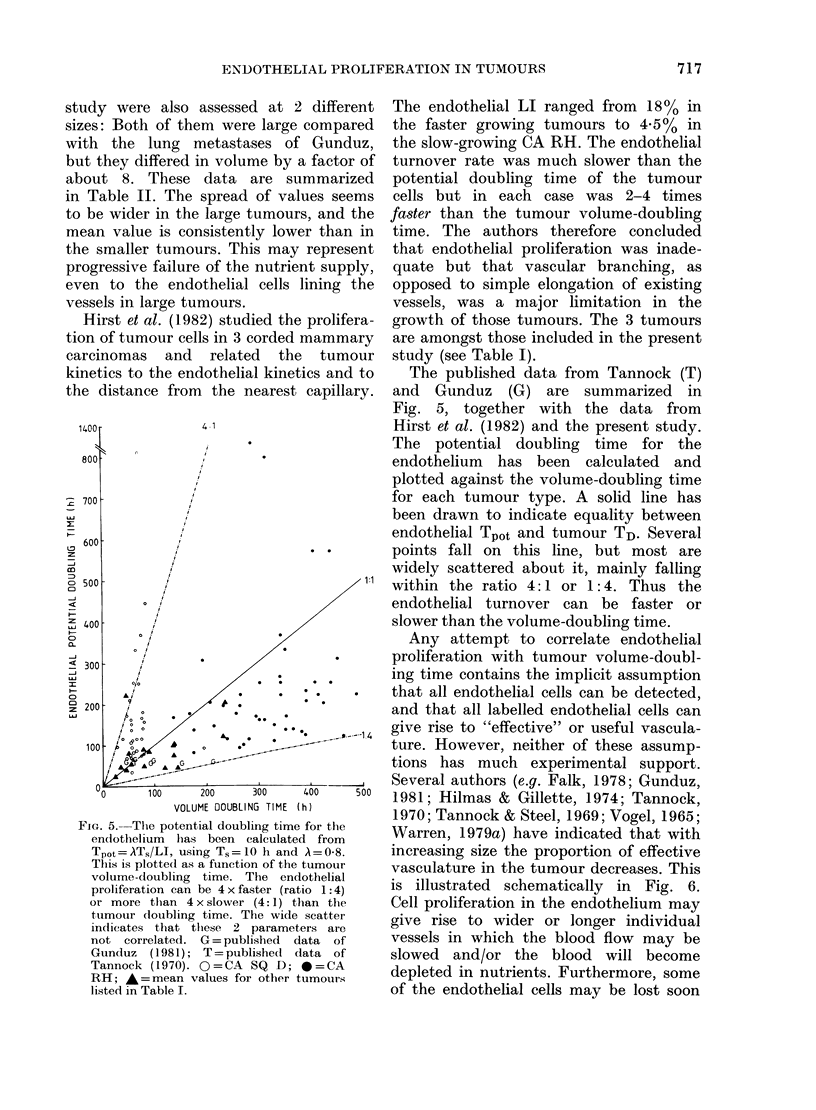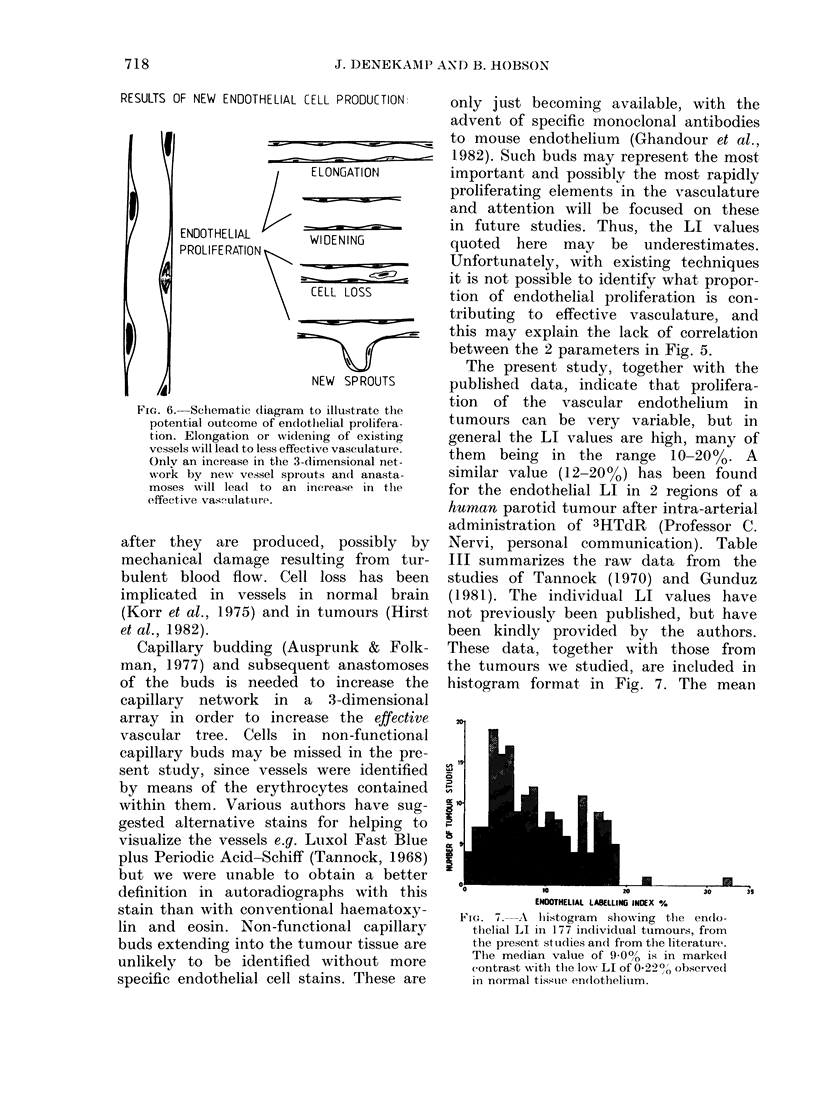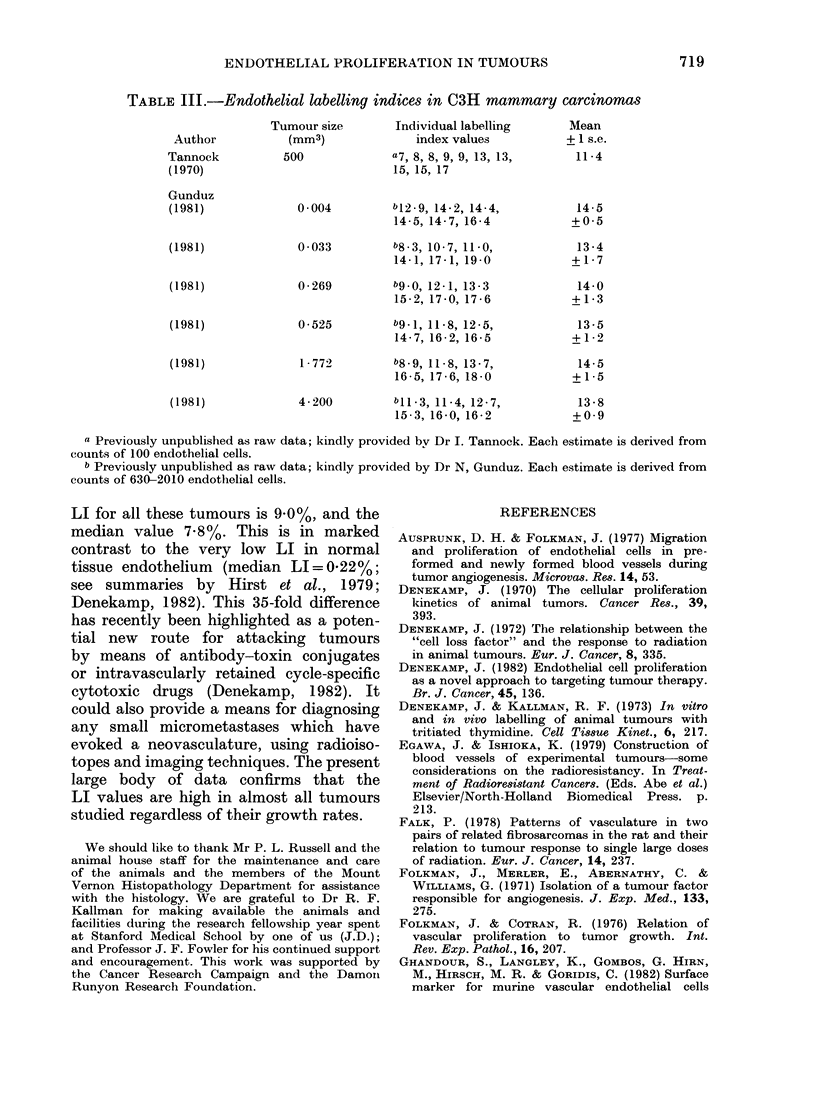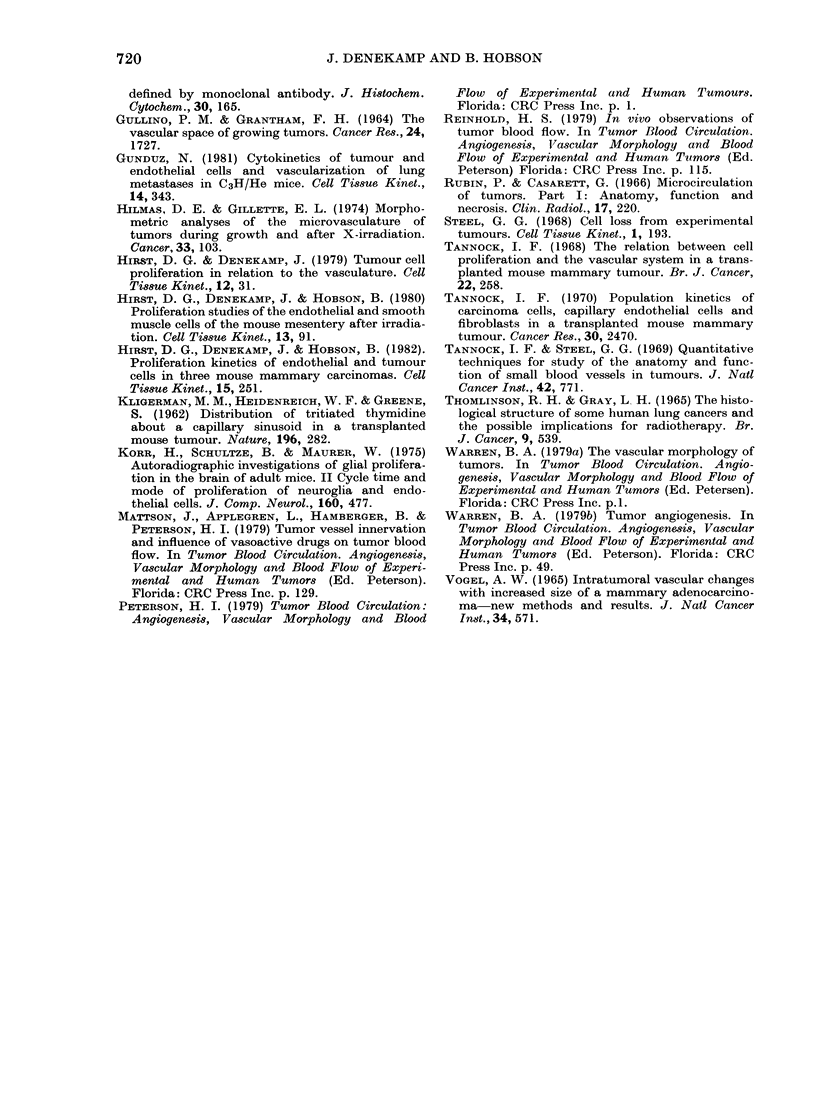# Endothelial-cell proliferation in experimental tumours.

**DOI:** 10.1038/bjc.1982.263

**Published:** 1982-11

**Authors:** J. Denekamp, B. Hobson

## Abstract

The proliferation characteristics of vascular endothelium have been studied in 131 individual experimental tumours, representing 18 transplanted tumour lines. The labelling index (LI) is high in most tumours, with a mean value of 0.9%, regardless of the growth rate of the tumours, or whether different tumour types are considered or individual tumours from within one line are studied in detail. A similar high LI value has been found by others for a human tumour. These high LI values may even underestimate the proliferation in new capillary buds. The high proliferative index of tumour endothelium is in marked contrast with the previously reported low 3HTdR uptake into normal tissue blood vessels. It seems likely that it is the type of new vessels formed that will influence tumour growth rates more than the simple rate of endothelial-cell proliferation. The large difference between the proliferation characteristics of tumour endothelium and normal tissue endothelium, recently identified as a possible approach for tumour therapy, has now been confirmed for a range of animal tumours and a human tumour.


*Br. J. Cancer* (1982) **46**, 711

### ENDOTHELIAL-CELL PROLIFERATION IN EXPERIMENTAL TUMOURS

J. DENEKAMP AND B. HOBSON

*From the Gray Laboratory of the Cancer Research Campaign, Mount Vernon Hospital, Northwood, Middlesex HA6 2RN*

Received 4 February 1982 Accepted 29 July 1982

**Summary.—The proliferation characteristics of vascular endothelium have been studied in 131 individual experimental tumours, representing 18 transplanted tumour lines. The labelling index (LI) is high in most tumours, with a mean value of 9·0%, regardless of the growth rate of the tumours, or whether different tumour types are considered or individual tumours from within one line are studied in detail. A similar high LI value has been found by others for a human tumour. These high LI values may even underestimate the proliferation in new capillary buds. The high proliferative index of tumour endothelium is in marked contrast with the previously reported low $^3$HTdR uptake into normal tissue blood vessels. It seems likely that it is the *type* of new vessels formed that will influence tumour growth rates more than the simple rate of endothelial-cell proliferation. The large difference between the proliferation characteristics of tumour endothelium and normal tissue endothelium, recently identified as a possible approach for tumour therapy, has now been confirmed for a range of animal tumours and a human tumour.**

THE VASCULAR NETWORK of tumours has long been known to differ from normal tissues, and to be inadequate in several aspects (for relevant reviews see Petersen, 1979). Tumour blood vessels are less well structured, more tortuous and permeable, less innervated and spaced further apart than the vessels in most normal tissues (Egawa & Ishioka, 1979; Falk, 1978; Gullino & Grantham, 1964; Hilmas & Gillette, 1974; Mattson *et al.*, 1979; Reinhold, 1979; Rubin & Casarett, 1966; Thomlinson & Gray, 1955; Vogel, 1965; Warren 1979*a*, *b*). A wide variety of of techniques has been used to study the morphology (both qualitatively and quantitatively) and function of tumour blood vessels (Peterson, 1979). These studies have indicated a considerable heterogeneity of structure and function amongst the individual vessels within 1 tumour, in the same tumour at different sizes, and amongst different tumours, whether of the same histological type or of differing histologies.

The disorganized vasculature of tumours is generally believed to result from the very rapid neovascularization that is evoked in tumours by the endogenous tumour angiogenesis factor, TAF (Folkman *et al.*, 1971; Folkman & Cotran, 1976). In spite of the production of many new vessels it is clear that vascular inadequacy can limit the proliferation of dependent tumour cells (Kligerman *et al.*, 1962; Tannock, 1968; Hirst & Denekamp, 1979; Hirst *et al.*, 1982). Tumour cells distant from blood vessels are thought to be radioresistant because of their reduced oxygenation (Thomlinson & Gray, 1955), and chemoresistant because of their nutritional deprivation.

In spite of the general interest in tumour blood vessels there have been only 3 studies of the proliferation rate of their component cells (Tannock, 1970; Gunduz, 1981; Hirst *et al.*, 1982). The scarcity of data probably results from the difficulty in recognizing endothelial cells in tumours because of the absence of endotheliumspecific stains. In addition, cyclic fluctuations in blood flow through vessels may result in an inhomogeneous distribution of radioactive tracers, so that the proliferation may be heterogeneous in different regions of the tumour (Tannock & Steel, 1969; Reinhold, 1979). Thus several limitations of the kinetic parameters that can be measured must be recognized at the outset, especially that the average values which can be obtained with standard cell kinetic techniques may not give any indication of regional variations. However, because of the shortage of information in this area and the potential clinical importance of such data (Denekamp, 1982) we have undertaken a study of thymidine uptake into the endothelium of a variety of different experimental tumours. Tumours of widely different growth rates have been studied and the relationship between growth rate, tumour-cell labelling and endothelial-cell labelling has been investigated.

### MATERIALS AND METHODS

The labelling experiments described in this paper were performed over the period 1965–81, but all the assessments of endothelial labelling indices have been made in 1980–81. Some of the details of tumour-cell proliferation have been published previously, but generally without reference to the vasculature. Table I lists the tumours in the order of their growth rates, together with some information about their origin, histology and previous publications. In general the tumours were transplanted s.c., by the simplest technique possible, and were measured at frequent intervals with Vernier calipers. At the chosen size (usually 7–10 mm mean diameter, unless otherwise stated) they were labelled by giving a single i.p. injection of 0·5–1 $\mu$Ci/g of low-specific activity tritiated thymidine (0·36–1·0 $\mu$Ci/mol). The animals were killed 0·5–2 h later; tumours were removed, bisected for fixation in formol saline, processed for histology and 4–5$\mu$m sections were cut. Autoradiographs were produced using Ilford K5 or Kodak NTB2 emulsion and were subsequently stained with haematoxylin and eosin.

Blood vessels were identified under the microscope by the presence of red blood cells in a space bounded by flattened endothelial cells. The vessels were easier to identify in carcinomata than in sarcomata, probably because of the more obvious differences between tumour and stromal cells in carcinomata, and possibly also because of a greater degree of shrinkage of fibrosarcomata during fixation. Two to eleven hundred endothelial cells were counted in most tumours but in 7 of the 131 tumours <100 endothelial cells could be identified. These were mainly the small tumours listed in Table II. The difficulty in finding large numbers of tumour endothelial cells has prevented us from classifying the vessels into different categories, either by size or position within the tumour.

The labelling index (L.I.) of the tumour cells was also scored by random scanning of the tumour sections. Approximately 2000 tumour cells were counted for each LI determination. A grain count of 3 grains above background was set as the detection limit for a labelled cell. Background levels were generally <1 grain per cell-sized area.

### RESULTS

One hundred and thirty-one individual tumours have been assessed. For some tumour types only 1 tumour has been studied; for others up to 53 tumours have been scored. Table I summarizes the growth rates and the LIs of endothelial cells and of tumour cells. The tumours varied widely in growth rates, with mean doubling times from 1 to 13 days. The mean values and the standard error ($\pm$1 s.e.) are indicated for the LIs where more than 1 tumour was sampled. A 9-fold range of mean LI values was observed for the tumour cells (7·1–60·5%). The range of LI values for endothelial cells was similar but they were generally lower (3·6–32·3%). The highest endothelial LI (32·3%) was observed in the sarcoma RIB5 in the Wistar rat, which had a doubling time of 24 h. The lowest mean endothelial LI was observed in the equally fast-growing lymphoma KHAA, but this was a tumour which grew by invasion into the surrounding tissue as a diffuse mass, rather than as a defined

TABLE I.—*Summary of proliferation kinetic data for tumours*

| Tumour | Animal | Histology | Vol. doubling time (h) | Tumour-cell cycle time (h) | Labelling index: Tumour cells (%) | Labelling index: Endothelium (%) | Potential doubling time: Tumour cells (h) | Potential doubling time: Endothelium (h) | Origins and references |
|---|---|---|---|---|---|---|---|---|---|
| RIB5 | Wistar rat | Anaplastic Sa. | 24 (30) | 13·5 | 30·0 (1) | 32·3 (1) | 24 | 25 | A, D |
| KHAA | C3H mouse | Lymphoma | 24 (10) | ~12[a] | 36·0±15·1 (6) | 3·6 (1) | 17 | 222 | B, F |
| KHJ | C3H mouse | Fibrosarcoma | 38 (4) | 16·5[a] | 33·7±3·5 (4) | 18·0 (1) | 24 | 44 | B, E, F |
| KHKK | BALB/C mouse | Rhabdomyosa. | 39 (6) | 15[a] | 60·5±4·8 (4) | 16·5 (1) | 12 | 48 | B, E, F |
| KHTD | C3H mouse | Fibrosarcoma | 48 (7) | — | 30·5±10·7 (3) | 16·7±2·0 (2) | 33 | 47 | B, E, F |
| CA TB | CBA/Ht mouse | Adenocarcinoma | 53 (3) | — | 17·1±0·6 (2) | 10·2±1·9 (8) | 47[b] | 78 | C, |
| CA AD | CBA/Ht mouse | Adenocarcinoma | 57 (5) | — | 20·2±2·2 (3) | 14·1±2·2 (7) | 40[b] | 57 | C, |
| KHLL | BALB/C mouse | Carcinoma | 62 (8) | 14[a] | 34·9±1·8 (2) | 9·5 (1) | 18 | 84 | B, E, F |
| CA SQD | WHt mouse | Squamous Ca. | 67 (33) | — | 34·6±1·9 (20) | 7·1±1·3 (33) | 23[b] | 113 | C, |
| CA BAC | CBA mouse | Adenocarcinoma | 79 (4) | — | 15·5±3·0 (2) | 9·0±2·2 (8) | 52[b] | 89 | C, |
| KHH | C3H mouse | Adenocarcinoma | 120 (10) | 19[a] | 27 (1) | 17·7 (1) | 24 | 45 | B, E, F, G, H |
| KHII | C3H mouse | Adenocarcinoma | 120 (10) | ~22[a] | 11·7±7·1 (2) | 7·7±2·0 (3) | 68 | 104 | B, E |
| KHHH | C3H mouse | Adenocarcinoma | 132 (7) | 21[a] | 13·9±2·6 (8) | 8·0±3·0 (5) | 52 | 100 | B, E, F |
| KHI | C3H mouse | Hepatoma | 138 (7) | 23[a] | 19·6 (1) | 10·5 (1) | 41[b] | 76 | B, E, F |
| KHU | C3H mouse | Carcinoma | 140 (5) | 15·5 | 25·0 (1) | 17·9 (1) | 22 | 45 | B, E, F, H |
| CA RHf | Wht mouse | Adenocarcinoma | 233 (3) | — | 18·9±1·7 (3) | 6·6±0·7 (3) | 42[b] | 121 | C |
| SA Sf | CBA/Ht mouse | Fibrosarcoma | 235 (2) | — | 7·1 (1) | 3·9±1·6 (3) | 113[b] | 205 | C |
| CA RH | WHt mouse | Adenocarcinoma | 319 (45) | 21·5 | 11·0±0·5 (25) | 4·5±0·3 (53) | 73[b] | 178 | C, H |

Figure in brackets indicate the number of animals contributing to the value.

[a] Denekamp—unpublished data. Derived from percent labelled mitoses curves.

[b] $T_{pot}=\lambda T_S/LI$: $T_S$ has been assumed to be 10 h and $\lambda=0\cdot8$ where measured values are not available.

Tumour origins

A—Hammersmith Hospital 1964–68.

B—Stanford Medical School 1968–69.

C—Gray Laboratory.

Publications

D—Denekamp, 1970.

E—Denekamp, 1972.

F—Denekamp & Kallman, 1973.

G—Hirst & Denekamp, 1979.

H—Hirst *et al.*, 1982.

TABLE II.—*Endothelial labelling indices in tumours of different sizes*

| | Small tumours (30–80 mm³) | | Large tumours (230–410 mm³) | |
|---|---|---|---|---|
| Tumour | LI (%) | Mean (%) | LI (%) | Mean (%) |
| CA AD | 12·8<br>16·7<br>18·0 | 15·8 | 4·4<br>14·5<br>18·5 | 12·5 |
| CA BAC | 6·0<br>8·2<br>18·6 | 10·9 | 3·3<br>7·6<br>10·3 | 7·1 |
| CA TB | 10·9<br>11·4<br>15·6 | 12·6 | 5·2<br>8·3<br>13·5 | 9·0 |

nodule. In this respect it differed from all the other tumours. There is no obvious trend in LI for either tumour cells or endothelium with increasing volume-doubling time (Table I).

These data are presented in more detail in Figs. 1–4. Fig. 1 shows the mean LI for the endothelial cells plotted as a function of the mean volume-doubling time, $T_D$. Points without error bars represent tumours in which only 1 sample was studied. The error bars on other points represent 1 s.e. An attempt has been made to fit these data by regression analysis of all the 131 individual values; the correlation coefficient is low ($r = 0{\cdot}48$).

FIG. 1.—The labelling index of tumour blood vessels plotted as a function of the volume-doubling time of the tumours at the size at killing (data from 131 tumours). Points without errors represent single tumour determinations. Error bars represent ± 1 s.e. There is no significant correlation ($r = 0{\cdot}48$).

FIG. 2.—The endothelial labelling index as a function of the tumour-cell labelling index for 89 tumours. Error bars represent 1 s.e. There is less variability in endothelial LI than in tumour-cell LI within each tumour type. In general the endothelial LI values are lower than those for tumour cells ($r = 0{\cdot}43$).

A straight line clearly does not fit the data well, particularly for tumours with doubling times below 5 days. If the endothelial LI values are plotted as a function of the reciprocal of $T_D$ (*i.e.* the rate of increase in tumour volume) a slightly higher, but still poor, correlation coefficient is obtained ($r = 0{\cdot}71$). This demonstrates that the dependence of the growth rate on the endothelial proliferation is very weak in widely differing tumour types.

Fig. 2 shows the endothelial LI values plotted as a function of the tumour-cell LI values. The dashed line represents equality between these 2 parameters. The endothelial LI values are generally lower than the corresponding tumour-cell LI values, indicating that endothelial proliferation is generally slower than tumour-cell proliferation. A linear regression analysis of the data, weighted according to the number of tumours contributing to each point, again gives a very low correlation coefficient.

Figs 3 and 4 show a similar analysis of the data obtained from the 2 tumours which have been studied in most detail. These are the moderately rapidly growing

FIG. 3.—Endothelial LI as a function of volume-doubling times for (A) 33 individual CA SQ D tumours ($r = -0 \cdot 1$) and for (B) 45 individual CA RH tumours ($r = -0 \cdot 17$). There is no significant correlation between these 2 parameters.

FIG. 4.—Endothelial labelling index as a function of tumour-cell LI for (A) 20 individual CA SQ D tumours ($r = 0 \cdot 38$) and for (B) 25 individual CA RH tumours ($r = 0 \cdot 1$). The endothelial LI values are generally lower than those for the tumour cells and there is no correlation between them.

CA SQ D and the very slow-growing CA RH. In these figures each point represents the value for an individual tumour, rather than the mean for a group of similar tumours. Since the type of tumour cell is now constant, correlations of endothelial labelling with other parameters should be more clearly distinguishable.

In Fig. 3 the endothelial LI values are plotted against individual tumour volume-doubling times, on the left for CA SQ D and on the right for CA RH. The scales have been adjusted by a factor of 2 for both axes since the faster growing CA SQ D tumours also tend to have higher values of endothelial LI. There is a wide scatter in the data. Linear regression analysis indicates very clearly that within each tumour type there is *no* correlation between these parameters ($r = -0{\cdot}10$ and $-0{\cdot}17$ respectively).

Fig. 4 shows the comparison of endothelial and of tumour-cell LI values within these 2 tumour types. The endothelial LI values are generally much lower than those for tumour cells, and most of the data points fall well below the dashed line which would indicate equality. The data are widely scattered in each panel and cannot be fitted by a linear regression analysis; correlation coefficients are again very low ($r = 0{\cdot}38$ and $0{\cdot}10$). Clearly the rate of tumour-cell proliferation in each individual tumour is not directly related to the rate of endothelial-cell proliferation in the tumour blood vessels responsible for supplying them with nutrients.

### DISCUSSION

The data presented in this paper show that the measured endothelial labelling in tumours is generally high and is not closely correlated with the tumour growth rate, whether tumours of identical origin or of widely differing histologies are considered. Tumours seem to be capable of evoking more or less rapid endothelial proliferation, but this is not directly related to the growth rate of the individual tumours. It may relate to tumour-cell-specific differences in the ability to produce tumour angiogenesis factor (TAF), but we have no measurements of TAF to support this.

The LI determinations have been made in enlarged capillaries and sinusoids, which may not be the most actively proliferating regions in the tumour vasculature and these values may therefore be underestimates.

In the first kinetic study of vascular endothelium. Tannock (1970) observed that the LI of the endothelium was much lower (11·4%) than the LI of the tumour cells (35%), and there was an even greater discrepancy if only the tumour cells adjacent to the capillary were counted (LI = 50%). The rapid cell-cycle time (13 h) and potential doubling time* (22 h) of the tumour cells were contrasted with the slower $T_{pot}$ of the endothelial cells (50–60 h). For 0·5 g tumours the endothelial $T_{pot}$ *matched* the volume-doubling time of the tumours. He concluded that "the rather different rates of proliferation of parenchymal and stromal cells may be a major cause of slowing of tumour growth", although at larger sizes he also implicated blood stasis as an important factor. The contribution of endothelial cells to the tumour vasculature from an external, rapidly proliferating pool of precursors was excluded on the basis of continuous labelling studies.

No further studies of the kinetics of tumour vasculature were published until the comprehensive study of lung metastases by Gunduz (1981). He measured the cell kinetic parameters of tumour cells and endothelium in small tumours (ranging from 0·004 to 4·2 mm³) using many lung nodules to obtain very large numbers of endothelial cells (600–2000 per tumour). This detailed study showed that endothelial LI was independent of tumour growth rate, *i.e.* similar to our present results. In some tumours the endothelial LI values were even *higher* than the LI values for adjacent tumour cells. He demonstrated that vascular proliferation was more than adequate for tumour growth, and was not the major reason for development of areas of necrosis. The vascular volume increased as the tumours grew, but the proportion of small "effective" capillaries fell from 99% in small tumours to only 38% in large tumours.

Three of the tumours in the present

* Potential doubling time, $T_{pot} = \lambda T_S/LI$ where $\lambda$ is a correction factor for the non-uniform age distribution of cells through the cell cycle (Steel, 1968).

study were also assessed at 2 different sizes: Both of them were large compared with the lung metastases of Gunduz, but they differed in volume by a factor of about 8. These data are summarized in Table II. The spread of values seems to be wider in the large tumours, and the mean value is consistently lower than in the smaller tumours. This may represent progressive failure of the nutrient supply, even to the endothelial cells lining the vessels in large tumours.

Hirst *et al.* (1982) studied the proliferation of tumour cells in 3 corded mammary carcinomas and related the tumour kinetics to the endothelial kinetics and to the distance from the nearest capillary.

FIG. 5.—The potential doubling time for the endothelium has been calculated from $T_{pot} = \lambda T_s/LI$, using $T_s = 10$ h and $\lambda = 0{\cdot}8$. This is plotted as a function of the tumour volume-doubling time. The endothelial proliferation can be 4 × faster (ratio 1:4) or more than 4 × slower (4:1) than the tumour doubling time. The wide scatter indicates that these 2 parameters are not correlated. G = published data of Gunduz (1981); T = published data of Tannock (1970). ○ = CA SQ D; ● = CA RH; ▲ = mean values for other tumours listed in Table I.

The endothelial LI ranged from 18% in the faster growing tumours to 4·5% in the slow-growing CA RH. The endothelial turnover rate was much slower than the potential doubling time of the tumour cells but in each case was 2–4 times *faster* than the tumour volume-doubling time. The authors therefore concluded that endothelial proliferation was inadequate but that vascular branching, as opposed to simple elongation of existing vessels, was a major limitation in the growth of those tumours. The 3 tumours are amongst those included in the present study (see Table I).

The published data from Tannock (T) and Gunduz (G) are summarized in Fig. 5, together with the data from Hirst *et al.* (1982) and the present study. The potential doubling time for the endothelium has been calculated and plotted against the volume-doubling time for each tumour type. A solid line has been drawn to indicate equality between endothelial $T_{pot}$ and tumour $T_D$. Several points fall on this line, but most are widely scattered about it, mainly falling within the ratio 4:1 or 1:4. Thus the endothelial turnover can be faster or slower than the volume-doubling time.

Any attempt to correlate endothelial proliferation with tumour volume-doubling time contains the implicit assumption that all endothelial cells can be detected, and that all labelled endothelial cells can give rise to "effective" or useful vasculature. However, neither of these assumptions has much experimental support. Several authors (*e.g.* Falk, 1978; Gunduz, 1981; Hilmas & Gillette, 1974; Tannock, 1970; Tannock & Steel, 1969; Vogel, 1965; Warren, 1979*a*) have indicated that with increasing size the proportion of effective vasculature in the tumour decreases. This is illustrated schematically in Fig. 6. Cell proliferation in the endothelium may give rise to wider or longer individual vessels in which the blood flow may be slowed and/or the blood will become depleted in nutrients. Furthermore, some of the endothelial cells may be lost soon

FIG. 6.—Schematic diagram to illustrate the potential outcome of endothelial proliferation. Elongation or widening of existing vessels will lead to less effective vasculature. Only an increase in the 3-dimensional network by new vessel sprouts and anastamoses will lead to an increase in the effective vasculature.

after they are produced, possibly by mechanical damage resulting from turbulent blood flow. Cell loss has been implicated in vessels in normal brain (Korr *et al.*, 1975) and in tumours (Hirst *et al.*, 1982).

Capillary budding (Ausprunk & Folkman, 1977) and subsequent anastomoses of the buds is needed to increase the capillary network in a 3-dimensional array in order to increase the *effective* vascular tree. Cells in non-functional capillary buds may be missed in the present study, since vessels were identified by means of the erythrocytes contained within them. Various authors have suggested alternative stains for helping to visualize the vessels *e.g.* Luxol Fast Blue plus Periodic Acid–Schiff (Tannock, 1968) but we were unable to obtain a better definition in autoradiographs with this stain than with conventional haematoxylin and eosin. Non-functional capillary buds extending into the tumour tissue are unlikely to be identified without more specific endothelial cell stains. These are only just becoming available, with the advent of specific monoclonal antibodies to mouse endothelium (Ghandour *et al.*, 1982). Such buds may represent the most important and possibly the most rapidly proliferating elements in the vasculature and attention will be focused on these in future studies. Thus, the LI values quoted here may be underestimates. Unfortunately, with existing techniques it is not possible to identify what proportion of endothelial proliferation is contributing to effective vasculature, and this may explain the lack of correlation between the 2 parameters in Fig. 5.

The present study, together with the published data, indicate that proliferation of the vascular endothelium in tumours can be very variable, but in general the LI values are high, many of them being in the range 10–20%. A similar value (12–20%) has been found for the endothelial LI in 2 regions of a *human* parotid tumour after intra-arterial administration of $^3$HTdR (Professor C. Nervi, personal communication). Table III summarizes the raw data from the studies of Tannock (1970) and Gunduz (1981). The individual LI values have not previously been published, but have been kindly provided by the authors. These data, together with those from the tumours we studied, are included in histogram format in Fig. 7. The mean

FIG. 7.—A histogram showing the endothelial LI in 177 individual tumours, from the present studies and from the literature. The median value of 9·0% is in marked contrast with the low LI of 0·22% observed in normal tissue endothelium.

TABLE III.—*Endothelial labelling indices in* C3H *mammary carcinomas*

| Author | Tumour size ($mm^3$) | Individual labelling index values | Mean ±1 s.e. |
|---|---|---|---|
| Tannock (1970) | 500 | [a]7, 8, 8, 9, 9, 13, 13, 15, 15, 17 | 11·4 |
| Gunduz (1981) | 0·004 | [b]12·9, 14·2, 14·4, 14·5, 14·7, 16·4 | 14·5 ±0·5 |
| (1981) | 0·033 | [b]8·3, 10·7, 11·0, 14·1, 17·1, 19·0 | 13·4 ±1·7 |
| (1981) | 0·269 | [b]9·0, 12·1, 13·3 15·2, 17·0, 17·6 | 14·0 ±1·3 |
| (1981) | 0·525 | [b]9·1, 11·8, 12·5, 14·7, 16·2, 16·5 | 13·5 ±1·2 |
| (1981) | 1·772 | [b]8·9, 11·8, 13·7, 16·5, 17·6, 18·0 | 14·5 ±1·5 |
| (1981) | 4·200 | [b]11·3, 11·4, 12·7, 15·3, 16·0, 16·2 | 13·8 ±0·9 |

[a] Previously unpublished as raw data; kindly provided by Dr I. Tannock. Each estimate is derived from counts of 100 endothelial cells.
[b] Previously unpublished as raw data; kindly provided by Dr N, Gunduz. Each estimate is derived from counts of 630–2010 endothelial cells.

LI for all these tumours is 9·0%, and the median value 7·8%. This is in marked contrast to the very low LI in normal tissue endothelium (median LI = 0·22%; see summaries by Hirst *et al.*, 1979; Denekamp, 1982). This 35-fold difference has recently been highlighted as a potential new route for attacking tumours by means of antibody–toxin conjugates or intravascularly retained cycle-specific cytotoxic drugs (Denekamp, 1982). It could also provide a means for diagnosing any small micrometastases which have evoked a neovasculature, using radioisotopes and imaging techniques. The present large body of data confirms that the LI values are high in almost all tumours studied regardless of their growth rates.

We should like to thank Mr P. L. Russell and the animal house staff for the maintenance and care of the animals and the members of the Mount Vernon Histopathology Department for assistance with the histology. We are grateful to Dr R. F. Kallman for making available the animals and facilities during the research fellowship year spent at Stanford Medical School by one of us (J.D.); and Professor J. F. Fowler for his continued support and encouragement. This work was supported by the Cancer Research Campaign and the Damon Runyon Research Foundation.

### REFERENCES

AUSPRUNK, D. H. & FOLKMAN, J. (1977) Migration and proliferation of endothelial cells in preformed and newly formed blood vessels during tumor angiogenesis. *Microvas. Res.* **14**, 53.

DENEKAMP, J. (1970) The cellular proliferation kinetics of animal tumors. *Cancer Res.*, **39**, 393.

DENEKAMP, J. (1972) The relationship between the "cell loss factor" and the response to radiation in animal tumours. *Eur. J. Cancer*, **8**, 335.

DENEKAMP, J. (1982) Endothelial cell proliferation as a novel approach to targeting tumour therapy. *Br. J. Cancer*, **45**, 136.

DENEKAMP, J. & KALLMAN, R. F. (1973) *In vitro* and *in vivo* labelling of animal tumours with tritiated thymidine. *Cell Tissue Kinet.*, **6**, 217.

EGAWA, J. & ISHIOKA, K. (1979) Construction of blood vessels of experimental tumours—some considerations on the radioresistancy. In *Treatment of Radioresistant Cancers.* (Eds. Abe *et al.*) Elsevier/North-Holland Biomedical Press. p. 213.

FALK, P. (1978) Patterns of vasculature in two pairs of related fibrosarcomas in the rat and their relation to tumour response to single large doses of radiation. *Eur. J. Cancer*, **14**, 237.

FOLKMAN, J., MERLER, E., ABERNATHY, C. & WILLIAMS, G. (1971) Isolation of a tumour factor responsible for angiogenesis. *J. Exp. Med.*, **133**, 275.

FOLKMAN, J. & COTRAN, R. (1976) Relation of vascular proliferation to tumor growth. *Int. Rev. Exp. Pathol.*, **16**, 207.

GHANDOUR, S., LANGLEY, K., GOMBOS, G. HIRN, M., HIRSCH, M. R. & GORIDIS, C. (1982) Surface marker for murine vascular endothelial cells defined by monoclonal antibody. *J. Histochem. Cytochem.*, **30**, 165.

GULLINO, P. M. & GRANTHAM, F. H. (1964) The vascular space of growing tumors. *Cancer Res.*, **24**, 1727.

GUNDUZ, N. (1981) Cytokinetics of tumour and endothelial cells and vascularization of lung metastases in C3H/He mice. *Cell Tissue Kinet.*, **14**, 343.

HILMAS, D. E. & GILLETTE, E. L. (1974) Morphometric analyses of the microvasculature of tumors during growth and after X-irradiation. *Cancer*, **33**, 103.

HIRST, D. G. & DENEKAMP, J. (1979) Tumour cell proliferation in relation to the vasculature. *Cell Tissue Kinet.*, **12**, 31.

HIRST, D. G., DENEKAMP, J. & HOBSON, B. (1980) Proliferation studies of the endothelial and smooth muscle cells of the mouse mesentery after irradiation. *Cell Tissue Kinet.*, **13**, 91.

HIRST, D. G., DENEKAMP, J. & HOBSON, B. (1982). Proliferation kinetics of endothelial and tumour cells in three mouse mammary carcinomas. *Cell Tissue Kinet.*, **15**, 251.

KLIGERMAN, M. M., HEIDENREICH, W. F. & GREENE, S. (1962) Distribution of tritiated thymidine about a capillary sinusoid in a transplanted mouse tumour. *Nature*, **196**, 282.

KORR, H., SCHULTZE, B. & MAURER, W. (1975) Autoradiographic investigations of glial proliferation in the brain of adult mice. II Cycle time and mode of proliferation of neuroglia and endothelial cells. *J. Comp. Neurol.*, **160**, 477.

MATTSON, J., APPLEGREN, L., HAMBERGER, B. & PETERSON, H. I. (1979) Tumor vessel innervation and influence of vasoactive drugs on tumor blood flow. In *Tumor Blood Circulation. Angiogenesis, Vascular Morphology and Blood Flow of Experimental and Human Tumors* (Ed. Peterson). Florida: CRC Press Inc. p. 129.

PETERSON, H. I. (1979) *Tumor Blood Circulation: Angiogenesis, Vascular Morphology and Blood Flow of Experimental and Human Tumours.* Florida: CRC Press Inc. p. 1.

REINHOLD, H. S. (1979) *In vivo* observations of tumor blood flow. In *Tumor Blood Circulation. Angiogenesis, Vascular Morphology and Blood Flow of Experimental and Human Tumors* (Ed. Peterson) Florida: CRC Press Inc. p. 115.

RUBIN, P. & CASARETT, G. (1966) Microcirculation of tumors. Part I: Anatomy, function and necrosis. *Clin. Radiol.*, **17**, 220.

STEEL, G. G. (1968) Cell loss from experimental tumours. *Cell Tissue Kinet.*, **1**, 193.

TANNOCK, I. F. (1968) The relation between cell proliferation and the vascular system in a transplanted mouse mammary tumour. *Br. J. Cancer*, **22**, 258.

TANNOCK, I. F. (1970) Population kinetics of carcinoma cells, capillary endothelial cells and fibroblasts in a transplanted mouse mammary tumour. *Cancer Res.*, **30**, 2470.

TANNOCK, I. F. & STEEL, G. G. (1969) Quantitative techniques for study of the anatomy and function of small blood vessels in tumours. *J. Natl Cancer Inst.*, **42**, 771.

THOMLINSON, R. H. & GRAY, L. H. (1965) The histological structure of some human lung cancers and the possible implications for radiotherapy. *Br. J. Cancer*, **9**, 539.

WARREN, B. A. (1979*a*) The vascular morphology of tumors. In *Tumor Blood Circulation. Angiogenesis, Vascular Morphology and Blood Flow of Experimental and Human Tumors* (Ed. Petersen). Florida: CRC Press Inc. p.1.

WARREN, B. A. (1979*b*) Tumor angiogenesis. In *Tumor Blood Circulation. Angiogenesis, Vascular Morphology and Blood Flow of Experimental and Human Tumors* (Ed. Peterson). Florida: CRC Press Inc. p. 49.

VOGEL, A. W. (1965) Intratumoral vascular changes with increased size of a mammary adenocarcinoma—new methods and results. *J. Natl Cancer Inst.*, **34**, 571.